# Climate change, disability, and water, sanitation and hygiene: A scoping review of evidence and interventions in low and middle-income countries

**DOI:** 10.1371/journal.pgph.0003676

**Published:** 2025-09-25

**Authors:** Jane Wilbur, Sari Kovats, Doug Ruuska, Shahpara Nawaz, Julian Natukunda

**Affiliations:** 1 International Centre for Evidence in Disability (ICED), London School of Hygiene & Tropical Medicine, London, United Kingdom; 2 Department of Public Health, Environments and Society, London School of Hygiene & Tropical Medicine, London, United Kingdom; 3 World Vision, GPO, Melbourne, Australia; 4 Environmental Health and WASH Research group, ICDDR, B, 68 Shaheed Tajuddin Ahmed Sharani Mohakhali, Dhaka, Bangladesh; 5 Nuffield Department of Medicine, Centre for Tropical Medicine and Global Health, University of Oxford, Oxford, United Kingdom; Emory University School of Medicine Credentialed through Emory Healthcare, UNITED STATES OF AMERICA

## Abstract

Climate hazards, including extreme weather events, undermine essential water, sanitation and hygiene (WASH) services, exacerbating health disparities in people with disabilities. Despite this, WASH policies and adaptation strategies often overlook the need for disability inclusive measures. The scoping review objectives are to 1) map the evidence on how climate risks affect WASH services and coping strategies in low-and middle-income countries, with a particular focus on people with disabilities; and 2) assess evidence for the effectiveness of climate-resilient WASH interventions, emphasising their impact on people with disabilities. Our review identified substantial data on women and girls, so the results reflect binary gender considerations. A systematic search of nine databases, including CINAHL Complete, Global Health, GreenFILE, and MEDLINE via PubMed, was conducted to identify peer-reviewed and grey literature using relevant keywords related to extreme weather and climate hazards, WASH (including menstrual health), disability, and evaluations. We included studies in English, published between January 1, 2000 and December 31, 2023. Data were extracted and analysed thematically. Twenty-two studies met the inclusion criteria. Only two papers evaluated climate-resilient WASH interventions (rainwater harvesting), and neither considered disability. Most papers examined people’s self-reported health impacts and experiences affected by drought-related water insecurity or WASH infrastructure damage due to extreme weather events. Data on the experiences of persons with disabilities were only included in two papers. They highlighted that persons with disabilities are disproportionately disadvantaged by climate-related damage to WASH infrastructure and emphasised the importance of social capital and social networks in supporting them when access to water is limited. Extreme weather events disrupt WASH services, yet evidence of the experiences and coping strategies of persons with disabilities remains extremely limited. This is a barrier to developing disability inclusive adaptation strategies. Evaluating climate-resilient WASH interventions is essential to enhance resilience and health equity for persons with disabilities.

## Introduction

### WASH and health

Access to water, sanitation, and hygiene (WASH) and related positive behaviours are essential for maintaining health and overall well-being [1]. Handwashing with soap and water can reduce acute respiratory infections—the world’s leading cause of morbidity and mortality—by 17% [2]. Improving access to WASH reduces childhood mortality from any cause by 17% and diarrhoea deaths by 45% [3]. Sanitation interventions can enhance the quality of life and prevent diseases. A study in unsewered, low-income areas of Maputo, Mozambique, found that people who used ‘high-quality’ shared latrines reported higher safety, privacy and mental health levels than users of ‘low-quality’ pit latrines [4]. High-quality latrines had pour-flush systems, concrete structures, and lockable doors, while low-quality ones had unlined pits and minimal privacy. Despite these physical and mental health benefits, nearly one in five people globally lack safely managed sanitation, and almost one in ten do not have access to safely managed water [5].

### Inequities in WASH

#### Disability.

Disability both results from and contributes to poverty, creating a cycle that is further reinforced by stigma and discrimination. A recent global study using the Multidimensional Poverty Index found that in 45% of the countries analysed, households with persons with disabilities experienced higher levels of poverty [6].

Inequities within WASH access are significant within and between countries. Individuals with disabilities - defined as those with long-term physical, mental, intellectual, or sensory impairments that, in interaction with various barriers, may hinder full and effective participation in society - often face limited or lower-quality access to WASH services, leading to disproportionate adverse health outcomes [7–9]. Despite often having poorer access, many people with disabilities have an even greater need for WASH services than those without disabilities. This is particularly true for individuals with conditions like incontinence, epilepsy, albinism, or skin diseases, as well as those who rely on orthotics, prostheses, or assistive devices that require regular cleaning [10–14].

#### Intersecting vulnerabilities.

Certain groups face challenges due to inadequate WASH services, which is exacerbating health inequities. These include women and girls, sexual and gender minorities, older adults, and persons with disabilities. Women and girls often bear the primary responsibility of collecting water from off-site sources, walking long distances, carrying heavy containers and sharing sanitation facilities, which can leave women and girls at risk of violence [5]. Disability increases with age, and older adults – who often share caregiving responsibilities with women and girls – encounter barriers to accessing WASH due to stigma and inaccessible services [15,16]. Sexual and gender minority groups experience discrimination in accessing WASH services, though there is a critical need for more research to understand and address the challenges fully [17].

Intersecting identities—such as gender, disability, age, wealth, ethnicity — can significantly amplify vulnerabilities and hinder access to WASH services. Individuals with multiple marginalised identities often face compounded barriers, further complicating their ability to meet essential WASH needs.

#### Climate change impacts on WASH services.

The threats of climate change to WASH services and behaviours are increasingly evident. Rising sea levels, extreme heat, changes to seasonal rainfall, flooding, and windstorms disrupt water availability, quality, and demand and sanitation services. Flooded sanitation facilities contaminate water supplies, and floods damage WASH infrastructure like water points, latrines, and sewerage systems [4,18]. These disruptions directly impact WASH behaviours that increase health risks: open defecation, unsafe water consumption, and long, often difficult journeys to collect water, with higher risk of heat injury or trauma from slips and falls. Menstrual health, handwashing, and personal hygiene practices are disrupted by lack of access to water, increasing health risks [19,20].

Many people with disabilities live in poverty, face insecure livelihoods, and encounter barriers to services [21]. This limits their ability to prepare for and adapt to climate hazards equally with others [22], while climate hazards further deepen existing inequities, such as limited access to WASH services and extreme poverty. Moreover, people with disabilities are often excluded from climate mitigation, adaptation, and response efforts [23,24],

leaving them without the benefits of these actions and potentially creating more disabling environments.

### Theoretical perspectives on climate change and WASH

How climate change is conceptualised with WASH influences stakeholders’ approaches to enhance climate resilience. Kohlitz et al. [25] documented three theoretical approaches for addressing climate change effects on WASH services: outcome vulnerability, contextual vulnerability, and resilience. Table 1 summarises approaches to linking climate change, adaptation, and WASH in a coherent framework.

**Table 1:** Theoretical perspectives on climate change and WASH.

Characteristics	Outcome vulnerability	Contextual vulnerability	Resilience
**Primary systems of interest**	Physical systems, particularly technologies	Social factors	Ecological, social-ecological and their interactions
**Timeframe of focus**	Near future (as far as models will allow).	Present	Long-term future
**Common analytical objectives**	Develop future climate scenarios, typically using models that project changes in the global climate and their impacts on physical systems like WASH infrastructure or water resources.	Understand who is least and most likely to cope with changes in environment and why. E.g., highlight the role of non-climatic drivers that exacerbate inequalities making certain social groups (i.e., persons with disabilities, gender minority groups, and low-income households) more susceptible to harm from climate change.	Understand how interactions within and between systems can withstand change or disturbance without shifting to an alternate, less functional state when thresholds are crossed, e.g., over-extraction of groundwater and rising sea levels can lead to saltwater infiltration. If natural recharge is insufficient, the aquifer may be become saline, compromising drinking water - signalling a shift in the system’s structure and function.
**Possible adaptation options**	Adaptation to reduce the risks or impacts of climate hazards, often called ‘climate proofing,’ such as elevating latrines above flood levels or improving technology management.	Enhance people’s adaptive capacity to navigate uncertainty better and adapt to increasingly hazardous environments. E.g., initiatives aimed at fostering agency and empowerment.	Develop adaptive governance structures and processes to help systems endure changes. Maladaptation is a critical concern—the unintended consequences of adaptation efforts that increase risks. E.g., increasing reliance on expensive technologies or centralising control over water resources can exacerbate existing inequalities and vulnerabilities, undermining the goal of building resilience.

Kohlitz et al. [25] explored how these theories were reflected in 33 papers documenting the WASH-climate change nexus. Most papers’ focus and efforts were predominantly grounded in outcome vulnerability, so they emphasised physical infrastructure. This is followed by contextual vulnerability and then resilience. There is growing attention to understanding climate-resilient WASH and how to operationalise it in different settings. The Sanitation and Water for All Climate Task Team [26] recently published a definition of climate-resilient WASH services that incorporates all three theoretical concepts for a holistic and comprehensive understanding (see S1 Text for working definitions).

Over the last 2.5 years, Water for Women - the Australian Government’s flagship WASH programme – has supported climate-resilient inclusive WASH projects and research in 16 Asian and Pacific countries. This work contributes essential evidence-based learning on the topic. Through recently published documents showcasing learning from fund partners, Water for Women highlights critical approaches for climate-resilient inclusive WASH delivery [27], captured in S2 Text for Water for Women’s Critical Approaches for Climate-Resilient Inclusive WASH Development. A core component emphasises the invaluable knowledge, capacities, and resilience that women and marginalised groups contribute, shaped by their lived experiences and cross-sector collaboration. Water for Women highlights that inclusive WASH is essential, stating that “climate-resilient WASH systems are only possible when gender equality, disability, and social inclusion principles are applied, ensuring all community members can adapt to and withstand climate events.”

### Scoping review objectives

Within this context, this scoping review has two objectives: to 1) map the evidence on how climate risks affect WASH services and coping strategies in low and middle income country populations, with a particular focus on people with disabilities; and 2) assess evidence for the effectiveness of climate-resilient WASH interventions, emphasising their impact on people with disabilities.

## Materials and methods

### Study design

This review followed the Joanna Briggs Institute methodology for scoping reviews [28]. A review protocol was registered online with OSF Registries (https://doi.org/10.17605/OSF.IO/SZCGB) and is reported in line with the PRISMA Extension for scoping reviews (S1 Checklist PRISMA Checklist). The working definitions applied in this manuscript are captured in S1 Text for working definitions.

### Search strategy

A preliminary search of PubMed was conducted to identify keywords and Medical Subject Headings (MESH) descriptors. These terms were then modified to suit the search in eight other databases (CINAHL Complete, Embase, Global Health; Web of Science; ECONLIT; DESASTRES, GreenFILE, and ERIC (Education Resources Information Centre). Four main concepts were used to develop the search strategy, i.e., weather or climate events, WASH, effectiveness, and disability (broad search terms provided in Table 2; detailed search strategy and keywords provided in S3 Text for the search strategy and key terms). Final search strings were peer-reviewed by academics experienced in the topics and the London School of Hygiene & Tropical Medicine (LSHTM) librarians.

**Table 2:** Broad search terms.

Key words	Broad search terms
Weather events (a)	“extreme weather” OR “climate change” OR “natural disaster*”
WASH terms (b)	“water service” OR “water access” OR “water suppl*” OR “hygiene” OR “sanitation” OR “WASH” OR “open defecation” OR “wastewater”” OR “menstrual and health” OR “menstrual and hygiene”, OR “handwashing”
Effectiveness terms (c)	“evaluation AND trial” OR “effectiveness AND trial” OR “outcome AND evaluation” OR “impact AND evaluation” OR “feasibility AND study” OR “summative AND assessment”
Disability (d)	“Disabled person*” OR person with disabilit* OR “handicapped person*” OR “Physical impair* OR “psychosocial” OR “cogniti* and disabilit*” OR “intellectual impair*” OR mental impair*, OR developmental disabili*,
Overall search strategies	a + b + c a + b + d [filters activated: English, from 2000/01/01 to 2023/12/3] Search strategy was rerun in May 2024

### Study selection and eligibility criteria

Two search strategies were developed: the first focused on studies examining the impact of climate risks, including extreme weather events, on WASH services and access for persons with disabilities, as well as associated coping strategies. The second aimed to identify studies evaluating the effectiveness of climate-resilient WASH interventions. The searches were limited to studies written in English, published between January 1, 2000 and December 31, 2023 and focused on low-and middle-income countries (LMICs) (using the World Bank classification), chosen for their heightened vulnerability to climate impacts and resource constraints. This time frame was selected due to the significant increase in research over the past two decades on climate change, WASH, and the growing emphasis on disability inclusion, aligning with global efforts toward climate resilience and sustainable development.

The initial search was conducted in July 2023 (covering articles from 2000 through 2022) and rerun in May 2024 (capturing articles published up to December 2023). Grey literature was searched using OPENGrey, WHO, AHRQ, BASE and Google Scholar to identify additional relevant literature. When an included article summarised findings from multiple primary studies, we manually searched for individual publications. Results were exported to EndNote X9 for de-duplication and then imported to Rayyan for screening. One reviewer screened the titles, while the abstract and full-text screening process was blinded and conducted independently by three reviewers. Disagreements were resolved by consensus. Eligible studies included participants with and without disabilities, provided they met the inclusion criteria in Table 3.

**Table 3:** Inclusion criteria.

Inclusion criteria	Exclusion criteria
Peer-reviewed research articles (all study designs, reviews) and grey literature Studies that reported weather or climate impacts on WASH services and behaviours OR papers that assessed the effectiveness of climate-resilient WASH interventions in community settings (e.g., shared handpumps/latrines) and within individual households Studies undertaken in LMICs Papers published (full texts available) between 2000 and 2023 and written in English	Focused on risks unrelated to the weather events such flood events from dam bursts Assessed WASH services for people in temporary accommodation following a disaster, including displacement camps Were commentaries, blogs, opinion pieces, not written in English language and were published before 2000

### Transparency statement on search strategy

During the later stages of review, we recognised that our initial search strategy had inadvertently excluded the specific terms “extreme heat,” “high temperatures,” and “cold temperatures.” To determine whether this omission affected our findings, we re‐ran the search, now including these terms across PubMed, CINAHL, GreenFILE, EconLit, and ERIC, and screened the top 20 Google Scholar results. No additional eligible studies were identified. As our original strategy already incorporated broader climate‐related terms (e.g., “climate change,” “extreme weather events”), which captured temperature‐related risks, we are confident that our conclusions are robust. Only four of the 22 included papers mentioned extreme heat among climate risks.

### Data extraction and analysis

The following data were extracted into Microsoft Excel for analysis: country, aims/purpose, study population and sample size, methods, intervention type, concept, duration of the intervention, how outcomes were measured, and key findings related to the review questions, including extended impacts on health outcomes. Due to the heterogeneous nature of the evidence gathered, applying a standardised reference scale to assess quality was impossible. This approach corresponds with guidance for conducting systematic scoping reviews, which states that, unlike systematic reviews, scoping reviews rarely formally evaluate the methodological quality of included papers [29].

## Results

Our research objectives and search strategy were centred on examining the impacts of climate risks to WASH services for persons with disabilities. However, during the data review process, we encountered substantial information addressing the effects on women and girls (not gender identities more broadly). As a result, our findings incorporate insights related to persons with disabilities and women and girls, while acknowledging that additional literature on the experiences of women and girls in relation to WASH and climate change may not have been captured by our specific search strategy.

### Study selection

**Fig 1:**
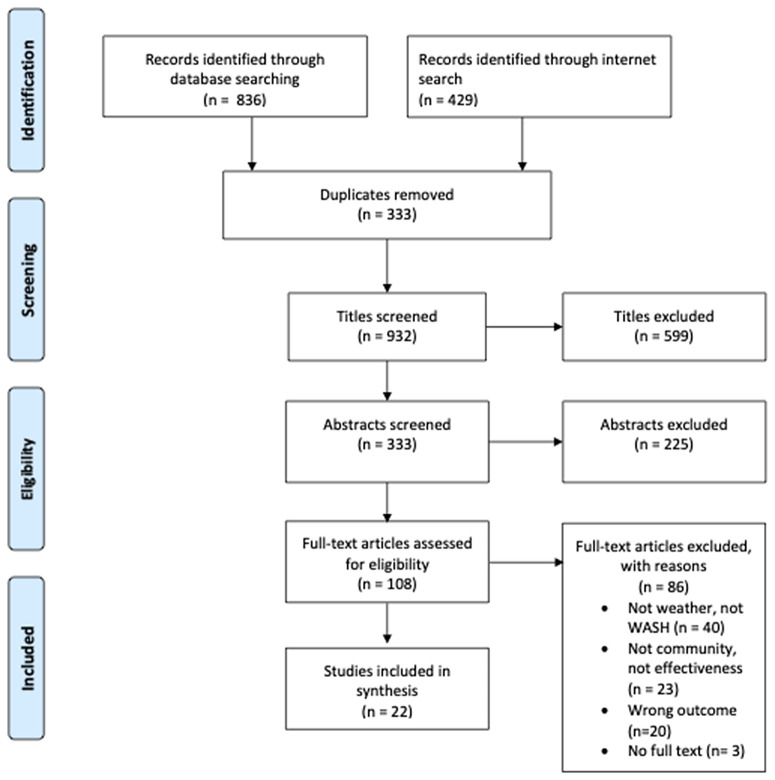
Search strategy with PRISMA flow diagram.

Twenty-two studies met our inclusion criteria (Fig 1 PRISMA flowchart). 1265 records were identified through electronic searches. After removing duplicates (n = 333), 824 records were excluded during the title and abstract screen, leaving 108 studies for the full-text screen. Following a full-text review, 86 studies were excluded, and 22 were selected for inclusion.

### Study characteristics

A summary of the characteristics of the included papers is presented in S1 Data for the characteristics of included papers. Regarding the theoretical approach, 50% (n = 11) of papers were grounded in outcome vulnerability, followed by 27% (n = 6) focused on outcome and contextual vulnerability. 14% (n = 3) drew on all three (outcome vulnerability, contextual vulnerability and resilience), and 9% (n = 2) considered contextual vulnerability independently. We did not find any studies that evaluated disability-inclusive climate-resilient WASH interventions, and two papers evaluated climate-resilient WASH at the community or household level. There was more evidence regarding the health impacts and lived experiences of persons dealing with climate-driven water insecurity or damage to WASH infrastructure from weather and climate extremes (91%; n = 20). The types of climate hazards considered were predominantly rainfall uncertainty and drought (86%; n = 19). Of these, four papers included data on extreme heat. Papers were from a wide range of countries, with Sub-Saharan Africa being the most represented region (36%; n = 8) and the least being East Asia and the Pacific (5%; n = 1). Most papers were published between 2020 and 2024 (82%; n = 18).

### Weather events and impacts on health, WASH services and behaviours

The publications examined a range of weather events presented in Table 4. Most focused on rainfall uncertainty and drought (n = 9) with four of those covering extreme heat, while others addressed combinations of rainfall uncertainty, drought, increased rainfall, and flooding (n = 7). Fewer studies considered heavy rainfall and flooding independently (n = 2), with even fewer investigating a broader scope, including rainfall uncertainty, drought, heavy rainfall, flooding, and climate-induced sea-level rise (n = 3). Only one paper exclusively explored sea-level rise. Most studies addressed the impact of weather events on water services alone (n = 6), and then equal attention was given to water and sanitation (n = 5), water and hygiene (n = 5), and water, sanitation, and hygiene (n = 5). The fewest papers considered sanitation and hygiene together (n = 1). Of the 22 papers, 13 included results about women and girls (none included other gender identities), and only two considered persons with disabilities.

**Table 4:** Overview of studies: climate risks/weather events, aspect of WASH, theoretical approaches, and key findings on women and girls, and disability.

Reference	Country and setting	Weather events	Water, Sanitation, or Hygiene	Theoretical approach: Outcome vulnerability, Contextual vulnerability, Resilience	Methods	Key results	Key results: women and girls	Key results: disability
Ahmed, F. et al. (2021)	Pakistan, South Punjab, Semi-arid region.	Extreme heat, rainfall uncertainty and drought.	W, H	Contextual vulnerability	Qualitative. Focus Group Discussions. In-depth interviews with women, men, female health workers, n = 35.	Health impacts: diarrhoea, lethargy, stress, dehydration. Coping strategies: increased open defecation, used mud and stones for anal cleansing, reduced handwashing and laundry.	Health impacts: lethargy, stress, dehydration, reduction in breastmilk and breastfeeding. Coping strategies: Walked further for water, collected water and did laundry in groups, older adults collected water with girls, mothers left children at home alone.	N/A
Amondo, E.I. et al. (2022)	Uganda, all regions, tropical and semi-arid.	Rainfall uncertainty and drought.	W	Contextual vulnerability	Quantitative, secondary data analysis, women and men, n = 22,469	Health impacts: headache, fever, weakness, coughing, abdominal pain and diarrhoea. Coping strategies: some used domestic rainwater harvesting.	Health impacts: higher rates of illness than men. Coping strategies: Walked further to water source, spent longer collecting, some used domestic rainwater harvesting.	N/A
Ashrafuzzaman, M. et al (2023)	Bangladesh, The Ganges Delta, tropical.	Rainfall uncertainty and drought. Increased rainfall, flooding and cyclones. Climate change induced sea level rise and saltwater intrusion.	W, S	Outcome vulnerability, Contextual vulnerability	Mixed methods, household survey, interviews, focus group discussions, case study.	Health: diarrhoea, dysentery, high blood pressure. Coping strategies: walked further for water, multiple use of water (deep tubewells, shallow wells, rainwater harvesting, unprotected surface water), pond sand filters, installing piped water, buying water, use unhygienic latrines, use pond water for cooking.	Health (maternal): hypertension, pre-eclampsia and respiratory infections. Coping strategies: not given.	N/A
Broyles, L.M.T. et al. (2022)	LMICs	Rainfall uncertainty and drought. Increased rainfall and flooding.	W	Outcome vulnerability, Contextual vulnerability	Systematic review (PRISMA guidelines), n = 67 papers.	Health impacts: Hypertension. Coping strategies: walk longer distances, use alternative sources, collect water at night to avoid queues, buy water (cost increases in dry season), and use poorer quality water if it’s closer to home, collect water from neighbours and kin.	N/A	N/A
Bukachi. S.A. et al. (2021)	Kenya, Kikui county, Semi-arid	Rainfall uncertainty and drought.	W	Contextual vulnerability	Qualitative, Focus Group Discussions (n = 10), In-depth Interviews (n = 85), Women, men.	Health impacts: none given. Coping strategies: Men identify people with water from within networks.	Health impacts: None given. Coping strategies: Collect and share water across networks based on reciprocity.	Health impacts: none given. Coping strategies: relied on friends, neighbours, church to collect water, based on moral obligation.
Carlton, E.J et al. (2014)	Ecuador, river basin, tropical.	Increased rainfall and flooding.	W, S, H	Outcome vulnerability, Contextual vulnerability	Quantitative, household survey, n = 19	Health impacts: Diarrhoea. Coping strategies: Treated drinking water.	N/A	N/A
Cassivi, A. (2022)	Malawi, southern region, semi-arid.	Extreme heat, rainfall uncertainty and drought.	W	Outcome vulnerability	Quantitative, household survey, n = 375. Systematic review (PRISMA guidelines), n = 20 papers	Health impacts: Diarrhoea. Coping strategies: Multiple use of water services, including informal rainwater harvesting to supplement a primary source.	N/A	N/A
Elgert, L. et al. (2016)	Guatemala, north-central, tropical.	Extreme heat, rainfall uncertainty and drought.	W, H	Outcome vulnerability	Qualitative, in-depth interview, observation, n = 12 households.	Health: not given. Coping strategies: Year-round rainwater harvesting at the household; supplemented with multiple trips to do laundry at public water points when rainwater levels dropped.	Health impacts: None given. Coping strategies: multiple trips to water points.	N/A
Foggit, A. (2021)	South Africa, Cape Town, Mediterranean	Rainfall uncertainty and drought.	W, S	Outcome vulnerability, Contextual vulnerability	Mixed-methods, primary and secondary data analysis (n = 311 video stories), online interviews and questionnaire (n = 39).	Health impacts: Psychological stress, water-related diseases (e.g., diarrhoea). Coping strategies: water rationing, drinking unsafe water, walking further for water.	Health impacts: psychological stress caused by constant fear of water shortages. Coping strategies: none given.	N/A
French, M.A. Et al. (2021)	Indonesia, Eastern region, tropical	Rainfall uncertainty and drought.	W, H	Outcome vulnerability	Quantitative, household surveys, soil and water sampling n = 2,764 people, 593 households.	Health impacts: diarrhoea, psychological stress. Coping strategies: use water bottle refill depot for drinking, multiple use of water service for personal and food hygiene.	N/A	N/A
Glasgow, L. & Mendes, J. (2020)	Grenada, Southern Grenadines, tropical.	Rainfall uncertainty and drought.	W, H	Outcome vulnerability	Quantitative, household survey, n = 20 households	Health: Diarrhoea. Coping strategies: using untreated rainwater for drinking, cooking, bathing, flushing toilets; collected water from neighbours.	N/A	N/A
Guo, D. et al (2019)	Tanzania, Buguruni (tropical savanna), Kilombero (tropical monsoon), and Kondoa (semi-arid) regions.	Rainfall uncertainty and drought. Increased rainfall and flooding.	W	Outcome vulnerability	Longitudinal cohort observational study, household surveys, water quality testing, observation, n = 636 households, 238 water sources.	Health: not given. Coping strategies: not given	N/A	N/A
Islam, M.M et al (2021)	Bangladesh, Khulna District, tropical.	Increased rainfall and flooding. Climate change induced sea level rise and saltwater intrusion.	W, H	Outcome vulnerability	Mixed-methods, household surveys and observation, n = 109 households.	Health impact: none given. Coping strategies: Used Pond water for personal hygiene and laundry, bought water for drinking and cooking, employed women to collect water from distant sources.	N/A	N/A
Iyer, R. & Pare Toe, L (2022)	Burkina Faso, Fada Gourma province. semi-arid.	Rainfall uncertainty and drought. Increased rainfall and flooding.	W, S, H	Outcome vulnerability, Contextual vulnerability, Resilience	Qualitative, participatory, interviews, focus group discussions.	Health: Mental fatigue. Coping strategies: prioritised water for food and livelihoods, Use toilet paper or wood instead of water for anal cleansing, bathe in toilets and use water to clean the toilet, open defecation and/or use neighbour’s toilet, dig new pits instead of emptying/reusing, prioritised building latrines because of inadequate shrub coverage for open defecation, build more robust toilets or on higher ground, barricade homesteads to protect home and latrine from flooding.	Health: not given. Coping strategy: Travel further for water, prioritised water for food and livelihoods.	N/A
Kohlitz, J. et al. (2023)	Burkina Faso, Fada Gourma province. semi-arid. Bangladesh, Satkhira district, tropical.	Rainfall uncertainty and drought. Increased rainfall and flooding. Climate change induced sea level rise and saltwater intrusion.	S, H	Outcome vulnerability, Contextual vulnerability, Resilience	Qualitative, observation, interviews, participatory methods.	Health impacts: not given. Coping strategies: open defecation, reduction in bathing.	Health impacts: Rashes, burns, urinary tract infections, blisters. Coping strategies: use saline water for menstrual health, walk further for water.	N/A
Kundu, A.K.(2023)	Bangladesh, Barguna Sadar Upazila, tropical.	Rainfall uncertainty and drought. Increased rainfall, flooding and cyclones.	W, S	Outcome vulnerability	Mixed methods, online survey, interviews.	Health: water related diseases. Coping strategies: multiple use of water (deep tubewells, rainwater harvesting, unprotected surface water), boil water before drinking.	Health: not given. Coping strategy: walked further for water.	N/A
de Duren. N.R. et al (2021)	Brazil, Rio de Janeiro, tropical.	Increased rainfall and flooding.	W, S	Outcome vulnerability	Qualitative, focus group discussions, observation, n = 80 participants in 88 upgraded favelas.	Health impacts: none given. Coping strategies: none given.	N/A	N/A
Marcus, H. et al. (2023)	Kenya, Lake Victoria Basin, tropical	Rainfall uncertainty and drought. Increased rainfall and flooding.	W, S	Outcome vulnerability	Qualitative, in-depth interviews, focus group discussions, observation. N = 96	Health impacts: Diarrhoea. Coping strategies: Multiple use of water services (bottled, rainwater harvesting), recycling water, solar/chlorine water treatment, open defecation, foregoing handwashing during drought, consuming herbs for gastrointestinal health, building ditches for flooding.	Health impacts: none given. Coping strategies: pooling savings before rains in anticipation of potential latrine reconstruction.	N/A
Mitu, K. et al. (2022)	Bangladesh, Cox’s Bazar, Tropical	Rainfall uncertainty and drought. Increased rainfall and flooding.	W, S, H	Outcome vulnerability, Contextual vulnerability	Qualitative, in-depth interviews, focus group discussions, key informant interviews (N = 57 adolescents, n = 6 key informants)	Health impacts: Anxiety and stress. Coping strategies: Water: Travelling long distances for water, rainwater harvesting, paying for drinking water, boys collect water and bathe in canals or public water points. Sanitation: open defecation	Health impacts: none given. Coping strategies: Adolescent girls were not allowed to collect water, reduction in bathing.	Health impacts: none given. Coping strategies: Traverse more difficult terrain to toilets.
Nayebare, J.G. et al. (2020)	Uganda, Kalungu District, tropical	Rainfall uncertainty and drought. Increased rainfall and flooding.	W, S, H	Outcome vulnerability	Mixed methods, household surveys, observations, interviews, focus group discussions.	Health impacts: e-coli, diarrhoea. Coping strategies: boiling drinking water.	N/A	N/A
Nuzhat, S. et al. (2023)	Bangladesh, Satkhira district, tropical	Extreme heat, rainfall uncertainty and drought. Increased rainfall, flooding and cyclones. Climate change induced sea level rise and saltwater intrusion. Extreme heat	W, S, H	Outcome vulnerability, Contextual vulnerability, Resilience	Mixed-methods, survey, participatory workshops, focus group discussions	Health: water related diseases. Coping strategies: Drink and use saline water, build toilets with concrete and brick using ceramic/porcelain tiles around concrete infrastructure, raise the height of toilet plinths or platforms of water sources, relocate sanitation and water facilities to higher ground.	Health: rashes, burns, urinary track infections, blisters from using salinized water for menstrual health. Coping mechanisms: use saline water for menstrual health. Walk further for water.	N/A
Wutich, A. et al. (2008)	Bolivia, central Bolivia, tropical	Rainfall uncertainty and drought.	W	Outcome vulnerability, Contextual vulnerability	Mixed-methods, surveys, in-depth interviews, observation, key informant interviews n = 72 household heads.	Health impacts: diarrhoea, dehydration, intestinal worms, stress. Coping strategies: drinking contaminated water, reducing bathing.	Health impacts: Stress. Coping strategies: paying for water.	N/A

Table 4 summarises what weather events each paper reports, how this impacts population health, and the coping strategies applied by the general population, women, girls, and people with disabilities. Coping strategies do not necessarily result in positive health outcomes. For instance, a coping strategy applied during water scarcity could be reducing handwashing, which could lead to increased infectious diseases.

### Rainfall uncertainty, drought, extreme heat

Eleven papers reported that rising temperatures, rainfall uncertainty, reduced rainfall, and drought conditions impact household water availability, including water reliability, quantity, and quality [30–40]. Studies from Pakistan, Southern Malawi, Guatemala and Bangladesh explicitly included data on rising temperatures [31–33,40]. For instance, in Bangladesh, participants reported a significant and perceptible rise in temperature over recent decades, with older adults noting that current levels of heat are greater than anything they have experienced in the past 30 years [31]. Ahmed et al. [32] noted that chronic water scarcity in Pakistan, especially during the extended dry season (covering seven months of the year), severely limits access to safe water. Responses to extreme heat include conserving water and reducing heat-exposed labour in Guatemala [40] and increasing reliance on multiple water sources and prioritise proximity over quality to avoid long walks in extreme heat in Southern Malawi [33]. Similarly, to cope with water scarcity in Grenada, Indonesia and Bolivia, people diversified their water sources, including collecting water from rivers, rainwater, shallow wells, and vendors [38,39,41]. In the same settings, people also requested water loans and borrowed or begged for water from neighbours as coping strategies [38,41].

Social capital and kinship networks are crucial in facilitating water-sharing and support in rural Kenya. Bukachi et al. [42] highlighted how these networks, influenced by gender roles, enable water subsidisation and reciprocity between families. Men typically identified a water source for the family, leveraging their networks, friendships, neighbours, and family relations, while women were responsible for collecting the water or working in exchange for it. People with disabilities who could not independently collect enough water relied on the moral obligation within social networks of friends, neighbours and the church for their water. People with disabilities were not expected to provide a service in return.

Water scarcity has led to significant shifts in water use priorities in various countries. In Grenada and Bolivia, people relied on untreated rain and surface water for drinking [38,41]. Four papers highlighted that households in Pakistan, Kenya, Bolivia, Bangladesh, and Burkina Faso prioritised water for food and livelihood security over personal and food hygiene activities—such as handwashing, laundry, cleaning plates, and baby feeding instruments [32,36,41,43]. Two studies from Pakistan and Burkina Faso reported a shift from using water for anal cleansing, a common practice among Muslims, to alternative materials such as paper, wood, mud, and pebbles [32,44].

Studies in six countries explored how rainfall uncertainty and drought compromised WASH access, contributing to adverse health impacts, including diarrhoea, abdominal pain, headache, dehydration, intestinal worms, and psychological stress [33,37–39,41,45].

In South Africa, Bolivia, and Uganda, waterborne diseases, dehydration, and headaches were reported and linked to consuming contaminated water [37,41,45]. For instance, Wutich et al. [41] documented that almost a quarter (23%) of households reported experiencing dehydration and intestinal issues (e.g., diarrhoea) because of consuming contaminated water in Bolivia. Declining mental health due to rainfall uncertainty and drought was reported in studies from Burkina Faso, Bolivia, Indonesia, Pakistan, South Africa, and Bangladesh [32,37,39,41,44,46]. In Bolivia, 92% of respondents reported experiencing fear related to water insecurity, with 65% experiencing this fear in the week leading up to interviews [41]. Heads of households described how these feelings of anxiety, worry and anger often led to family conflicts and arguments.

Water scarcity and rising temperatures have significant gendered impacts, with women often bearing a heavier burden. In Burkina Faso, women reported that their work burden increased during water shortages as they had to manage available water for cooking, cleaning, laundry, and the kitchen garden [44]. Foggitt [37] reported that in South Africa, women were particularly affected by the constant fear of water shortages. Ashrafuzzaman et al. [30] reported that both men and women travelled further to collect water in Bangladesh. However, several studies from Pakistan, Uganda, Guatemala, and Bangladesh reported that women and girls were primarily responsible for water collection, often spending more time, covering greater distances, standing in longer queues, and making multiple trips [32,40,45,47].

Water collection under high temperatures increases fatigue and physical strain for women who are mostly responsible for this task. In southern Malawi, respondents frequently highlighted that women suffer exhaustion during water-fetching tasks, especially in the dry season [33]. In South Punjab, Pakistan, extreme summer temperatures intensify dehydration, fatigue, and the risk of kidney issues, while also leading to reduced lactation due to exhaustion and limited hydration [32]. In this setting, women were more stressed and lethargic, especially when sick or menstruating; dehydration was common, and this further affected infant care and feeding practices [32]. Additionally, women and girls faced harassment when collecting water, perpetrated by men, boys and other women queuing for water, were reported in Pakistan, Guatemala, and Bangladesh [32,40,47]. In South Punjab, Pakistan, water insecurity increased women’s and girls’ vulnerability to harassment and gender- and caste-based violence, including sextortion [32].

### Increased rainfall, flooding and cyclones

Heavy rainfall events and flooding have a detrimental impact on water quality. Flooding in Uganda, Tanzania, and Ecuador led to wells collapsing [34–36,48]. This introduced built-up bacteria and other harmful organisms (pathogens) directly into surface water used for drinking and bathing (Uganda, Tanzania, Ecuador) [35,48,49]. This was particularly concentrated when dry periods, during which pathogens had built up in the environment, were followed by heavy rainfall, resulting in a significantly increased risk of E. coli in water and diarrhoea transmission, as reported in studies from Uganda and Ecuador [35,49].

People in various countries have adopted different strategies to cope with water scarcity and contamination. In Uganda, Kenya, Bangladesh, and Ecuador, authors reported that people treat drinking water by filtering and boiling it, using solar water disinfection, and adding chlorine [35,36,47,49]. Other coping strategies include paying for drinking water and storing and recycling water. In Kenya and Bolivia, the most common approach was constructing rainwater harvesting systems at homes, which were used for drinking (if treated), bathing, laundry, and cooking [36,46].

Extreme weather has caused significant damage to sanitation infrastructure. Six studies from Bangladesh, Kenya, and Burkina Faso reported that heavy rains, flooding, and cyclones damaged latrine infrastructure, leading to waterlogging and overflowing latrines and drainage systems [30,31,36,44,46,47]. For instance, Marcus highlighted how heavy rains lead to pit latrine damage and collapse in Kenya’s Lake Victoria Basin [36]. In Rio de Janeiro, Libertun de Duren et al. [50] found that sewerage systems overflowed during heavy rains, but this was also caused by population growth overtaking the capacity of the sanitation system. In Cox’s Bazar, Bangladesh, Mitu et al. [46] reported that heavy rains, flash floods, and landslides caused waterlogging as drainage systems were not regularly cleared. The authors also noted that adolescents with disabilities living in long-term Rohingya refugee settlements faced additional challenges reaching latrines because heavy rains damaged roads and paths [52]. Nuzhat et al. noted that women did not want to use damaged latrines that were dark and lacked privacy in Bangladesh [31].

Weather events and disasters have had lasting effects on sanitation infrastructure and behaviours. Studies from Bangladesh and Burkina Faso reported that many latrines were often damaged from previous weather events, so they could not withstand the latest incident [31,44]. Sanitation was deprioritised for several reasons: 1) the recurring need to rebuild damaged latrines, causing mental fatigue and related costs after a weather event; 2) the inability to reach the local market to buy materials to rebuild the latrine because floods damage the roads, 3) a focus on rebuilding the homestead or food and livelihood security [31,44]. Iyer and Toe [44] highlight that in Burkina Faso, funding from the local government for sanitation is insufficient, leading to the construction of makeshift latrines that are easily damaged in weather events. Nor do they provide funding for retrofitting damaged latrines.

Two studies from Bangladesh reported continued use of unhygienic latrines [30,31], increased rates of open defecation [30], and using neighbours’ latrines [30]. Similarly, in Burkina Faso, people reported both increased open defecation and use of neighbours’ latrines [44]. Due to impacts on sanitation services and behaviours, participants in two studies from Bangladesh reported increased waterborne diseases, including diarrhoea and skin conditions [31,47]. A study in Bangladesh reported a 33% increase in diarrhoeal diseases in the three months after the event, associated with an average loss of 18 working days per person [47].

Response measures have been implemented in various contexts to mitigate the impacts of weather events [31,36,44]. People in Kenya built ditches and dams around houses for flood control, saved for latrine reconstruction ahead of the rainy season, constructed latrines in higher ground to increase resistance to flooding, and planted trees in household yards to improve soil water retention [36]. In Burkina Faso, some people built flood defences around their homesteads for protection [44]. Households who could afford to do so built stronger, more flood-proof latrines and used more durable materials such as strong cement pit liners and concrete slabs (Bangladesh, Burkina Faso) [31,44]; some relocated latrines to higher ground or raised the height of the latrine plinths above flood levels (Kenya, Burkina Faso) [36,44].

Heavy rainfall, flooding, and cyclones have had significant impacts on the ability of women and girls to manage menstruation in Bangladesh. Nuzhat et al. [31] reported that damage to latrines hindered people’s ability to change menstrual materials privately, while the heavy rains and flooding made it challenging to clean and dry reusable materials. Many individuals were forced to evacuate their homes without menstrual materials and were unable to buy replacements as shops were closed during weather events. Additionally, cyclone shelters lacked adequate water, sanitation, and hygiene services to support menstrual health.

### Sea level rise and saltwater intrusion

The impacts of sea level rise and saltwater intrusion were documented in three studies [30,31,34]. Saline intrusion into groundwater, shallow wells, and surface water has been documented in subtropical and tropical floodplain rivers, coastal rivers, and oceanic islands due to climate and non-climate causes [30,31,34]. In Bangladesh, this reduced water is used for drinking, cooking, and agriculture all year round [30,31]. Coping strategies have included using multiple water sources, primarily rainwater harvesting and deep tubewells. Most households used rainwater to drink when available (two to three months a year) and then shifted to salinised pond water [30]. The pond water was primarily used for cooking; some used community-level pond sand filtration, even though this does not remove salt. Those who could afford it bought water from Reverse Osmosis plants provided by the government and Non-Government Organisations or installed piped water in their homes in Bangladesh [30,31,51]. Yet, Ashrafuzzaman et al. [30] noted that pond sand filtration was rarely delivered in collaboration with community members in remote rural areas.

Reducing freshwater sources and supply led to greater dependence on highly saline water for domestic use, which increased health issues. In Bangladesh, reported health impacts of consuming highly saline water included high blood pressure, increased rates of hypertension, pre-eclampsia, and respiratory infections [30]. Additionally, women and girls who bathed in and washed reusable menstrual materials in water with high saline content reported burns, rashes, blisters, and urinary tract infections [31].

### Effectiveness of climate-resilient WASH at the household and community levels

There is minimal evidence regarding the effectiveness of climate-resilient WASH services at the household and community levels. Only two papers explored this, and both focused on rainwater harvesting technology (Table 5). No papers explored disability or efforts to maintain positive WASH behaviours (e.g., safe water management, handwashing with soap and water at critical times, faecal management and disposal, food hygiene and menstrual hygiene) before, during or after a climatic event.

**Table 5:** Studies on the effectiveness of climate-resilient WASH.

Study	Study aim	Study site and methodology	Technology	Key findings
Elgert, L. et al. (2016)	Evaluate if rainwater harvesting has improved water security in a peri-urban village in Guatemala and draws implications from this project for expansion to the region and beyond.	Peri-urban village in Guatemala. Qualitative: interviews and observation of rainwater harvesting technology, 12 households.	Household rainwater harvesting	Increased household water security and a reduction in time and energy expended by women collecting water from public water points. However, some negative impacts regarding cost, water quality.
Glasgow, L. & Mendes, J. (2020)	Assess the knowledge, attitudes and practices about household rainwater harvesting.	Carricoao, Grenada. Quantitative: survey of 20 households in 15 communities.	Household rainwater harvesting	Increased water security but some concerns about water quality associated with poor maintenance.

Elgert et al. [40] explored whether government-sponsored household rainwater harvesting improved water security (quantity, quality, and access) in 12 households in a Guatemalan peri-urban village and the potential to expand this approach regionally and beyond. Glasgow et al. [45] assessed respondents’ knowledge, attitudes, and practices using household rainwater harvesting systems in 20 homes in Carricoao, Grenada.

Both studies reported increased water quantity. Respondents in Elgert’s study noted using rainwater for almost six months of the year, meaning family members (primarily women) reduced the number of trips made to the public water points compared to those without the rainwater harvesting system [40]. Half of the participants in Glasgow et al.’s study reported water availability throughout the year [38]. In Guatemala, rainwater was primarily used for drinking and cooking, whilst laundry was done at public water points to conserve rainwater [53]. In Carricoao, respondents used rainwater for drinking, cooking, bathing, laundry and cleaning [38]. Individual household water treatment practices were not gathered in Elgert et al.’s study, but the authors recommended boiling water before consumption [40]. Glasgow et al.’s sample respondents reported that just over half treated water before drinking by adding bleach chlorine tablets and using small fish to consume insects [38]. No data on water-related or borne diseases was gathered in either study.

Both studies concluded that rainwater harvesting systems could increase water security but that regular operation and maintenance are critical. Additionally, Glasgow et al. highlighted the importance of educational campaigns to improve water treatment practices before consumption [38].

## Discussion

We found limited evidence on how climate risks, including weather events, affect WASH services and access for persons with disabilities, despite existing evidence on the impacts of and coping strategies for climate-driven extreme weather events. Additionally, there is limited data on the effectiveness of climate-resilient WASH interventions, with only two studies evaluating rainwater harvesting. Data on people with disabilities was found in only two studies: one in rural Kenya, where social networks based on moral obligation facilitated water access for those unable to collect it independently, and another in Cox’s Bazar, Bangladesh, where heavy rains, floods, and landslides worsened latrine accessibility for people with disabilities. This review highlights a significant evidence gap regarding the impact of climate-driven extreme weather events on the WASH experiences of people with disabilities, their coping strategies, and the related effects on their health and well-being. It also underscores their exclusion from response measures and strategies.

### Outcome vulnerability – physical systems, particularly technologies

#### Climate impacts on WASH services and health outcomes.

Most papers in our review focused on outcome vulnerability. However, findings often highlighted the cascading effects of climate impacts on WASH systems and technologies, including their influence on behaviours, health, and well-being. Participants frequently self-reported health impacts, underscoring the fragility of WASH systems and the widespread consequences of disruptions.

After climate-driven extreme weather events, many people adopted coping strategies such as consuming untreated water, diversifying water sources (e.g., using rain or pond water for cooking and hygiene), and reducing household water use. These strategies, common in many LMIC settings [4,52,53], demonstrate that access to diverse, high-quality water sources close to households can enhance resilience. Climate-resilient water systems must ensure continuity and adaptability across these sources while maintaining quality and accessibility.

Limited water resources were often prioritised for agriculture and livelihoods over sanitation and hygiene. This trade-off led to increased open defecation and reduced hygiene practices, such as handwashing, cleaning feeding utensils, laundry, and menstrual health management, all of which have serious health implications [32,36,41,43]. Notably, no data were found on the menstrual health experiences of women and girls with disabilities. While evidence underscores the importance of menstrual health, particularly in crises, the intersection of menstrual health, disability, and climate change remains unexplored [54–57]. Addressing these challenges requires prioritisation, as neglecting them will only deepen menstrual health inequities for people with disabilities.

Health impacts from climate-related events were reported across various settings. Increased rates of illnesses such as diarrhoea, abdominal pain, headaches, dehydration, intestinal worms (linked to rainfall variability, drought, extreme temperatures, flooding, and cyclones), and other conditions like high blood pressure, respiratory infections, and rashes (associated with saltwater intrusion) were frequently documented. Diarrhoeal disease, a leading cause of death and malnutrition in children under five [58], disproportionately affects children with disabilities, who experience twice the rate of severe diarrhoea compared to their counterparts without disabilities [59]. However, none of the reviewed studies that reported instances of diarrheal disease accounted for disability, revealing a critical gap given the health inequity experienced by persons with disabilities.

The disruptions to WASH services also negatively impacted mental health and well-being. Our review found evidence linking these disruptions to declines in mental health, aligning with expanding research on climate change and mental health. For instance, Charlson et al. [49] reported associations between climate-related exposures (e.g., rainfall variability, droughts, floods) and poor mental health outcomes such as distress, hospitalisations, and suicide, especially among individuals with pre-existing mental health conditions. Similarly, Wahid et al. [60] found that floods in Bangladesh significantly increased depression and anxiety, with higher odds for people with physical disabilities. Despite these findings, our review identified no further data on the intersection of disability, climate change, and mental health, highlighting an urgent need for research.

#### Coping strategies.

Our review identified various coping strategies aimed at increasing resilience to climate-driven extreme weather events. However, most of these strategies impose costs on households, likely exacerbating disparities, as only higher-income households can afford to ‘adapt.’ These coping strategies include pond sand filtration, reverse osmosis, purchasing water, installing piped water systems, constructing flood defences around homesteads, and building latrines with more durable materials on higher ground.

Affordability remains a significant barrier, particularly for persons with disabilities, who often live in greater financial poverty compared to those without disabilities. This underscores the need for social protection measures to ensure equitable access to these critical interventions and to prevent extreme events from pushing already vulnerable households further into poverty. WASH policies must specifically target persons with disabilities to address these inequities effectively.

#### Evaluations.

There was no evidence on the effectiveness of climate-resilient WASH interventions in terms of outcomes for persons with disabilities. The two papers in our review focused on rainwater harvesting, noting limited water treatment practices but not examining health outcomes or the maintenance of positive WASH behaviours. Evaluations of climate-resilient WASH services should comprehensively assess technologies used, user behaviours, and system maintenance practices throughout seasonal peaks in rainfall and during extreme weather [52]. Importantly, these evaluations must consider disability to ensure that interventions are inclusive and address the specific needs and challenges faced by persons with disabilities.

### Contextual vulnerability – social factors

#### Social capital and kinship networks.

Our review highlights the importance of social capital and kinship networks during water scarcity. However, people with disabilities are particularly vulnerable during droughts, as they often rely on the moral obligation of social networks to collect water. This reliance may decline as the situation worsens for everyone. Such vulnerability to growing inequalities underscores the need for targeted support mechanisms to ensure access to water for persons with disabilities during droughts.

Kinship and social capital have historically been crucial in distributing finite resources, and they are also seen as potential strategies for building resilience to climate change [61,62]. While social capital is essential for immediate survival, further research is needed to understand its broader effects on climate change adaptation. By exploring these coping mechanisms and their limitations, more effective strategies can be developed, such as promoting sustainable water management practices, strengthening social support networks, and providing targeted assistance for populations vulnerable to exclusion.

#### Gender and disability inclusion in climate-related WASH.

The intersection of climate change, gender, and disability remains underexplored in research and practice. While over half of the studies addressed the impacts of climate change on women and girls, evidence related to people with disabilities remains scarce. This pattern is also evident in studies assessing disability inclusion in WASH-related policies in Nepal, Bangladesh, and Cambodia, where gender received more attention than disability [63,64]. Across these policy analyses, disability was significantly less prioritised than gender. Both women and people with disabilities are vulnerable to climate change risks, but when these identities intersect, the compounded effects of marginalisation deepen their exposure to climate impacts. Future research must increase focus on both groups, ensuring that studies prioritise the experiences of women and girls with disabilities, as their intersecting identities exacerbate poverty and exclusion.

#### Nuanced barriers for persons with disabilities.

It is essential to recognise that disability is not a homogeneous group when assessing access to WASH services. Two papers specifically included results about persons with disabilities. However, one treated this group homogeneously, with limited consideration of specific impairments or gender, while the other focused only on physical accessibility to WASH services for individuals with sensory or physical disabilities [42,46]. A growing body of evidence highlights the importance of understanding how intersecting identities shape WASH experiences [9,16,65]. For instance, nuanced barriers faced by women and men with disabilities of different ages and impairment types are better documented, enabling the design of more tailored solutions [66]. These insights must now be integrated into climate change adaptation and resilience efforts for WASH services.

### Resilience - Ecological, social-ecological and their interactions

In the theoretical perspectives on climate change and WASH applied in this study, resilience focuses on ecological and socio-ecological systems, examining how these systems can endure change without degrading into less functional states. To enhance system resilience and support long-term adaptation, efforts must include establishing effective governance structures and processes [67]. Some papers in our review touched on governance, but this was primarily to indicate a lack of response measures and strategies in remote, rural areas of Bangladesh [30] or inadequate funding for household sanitation in Burkina Faso [44].

Effective climate risk management requires integrating traditional knowledge with climate science into existing governance systems while ensuring that diverse voices are included in decision-making [27]. Emerging examples illustrate these principles in practice. In Nepal, SNV trained key stakeholders to use nationally endorsed tools such as the WASH Management Information System and Climate-Resilient Water Safety Plans [68]. This approach enables local governments to prioritise water supply upgrades based on climate and disaster impact data, embedding climate resilience into standard procedures. This initiative is institutionalising climate risk management in WASH service provision by leveraging existing systems and building local capacity. In Papua New Guinea, WaterAid adopted a participatory dialogue approach to empower women and people with disabilities in WASH decision-making. By raising awareness of rights and promoting diverse representation in local governance, the project fostered a community-wide commitment to inclusive WASH practices [69].

### Emerging shifts in the emphasis on theoretical perspectives in climate-resilient WASH

Most papers in our review focused on outcome and contextual vulnerability, highlighting the impacts and pathways through which vulnerable groups are disadvantaged. This aligns with Kholitz’s 2017 review of 33 studies, which found that 79% predominantly used the outcome vulnerability perspective [25]. However, our findings reveal a broader distribution across all three theoretical perspectives—outcome, contextual, and resilience—used independently or in combination. This shift suggests a more holistic discourse on climate change and WASH, consistent with recent definitions of climate-resilient WASH services and Water for Women’s critical approaches to inclusive WASH development [26,27]. Resilience, therefore, is not purely technical but is deeply shaped by social factors that influence how communities experience and respond to climate impacts.

As efforts to operationalise climate-resilient WASH frameworks advance, rigorous evaluations must account for all dimensions of climate resilience across these theoretical perspectives. Data disaggregated by disability, gender, and other vulnerable groups is essential to ensure that interventions address existing inequalities rather than exacerbating them in the face of climate impacts.

### Strengths and limitations of review

This study employs a robust conceptual framework, providing a structured approach to organising and analysing the evidence, identifying gaps in the literature, and drawing connections between various factors, enhancing the overall understanding of the topic. Another key strength is its focus on vulnerabilities, using a disability lens. Notably, it highlights the risks faced by women and girls with and without disabilities, which aligns with the growing emphasis on gender considerations in climate-health research. By integrating these perspectives, the study offers a broader understanding of vulnerabilities and resilience strategies within WASH services.

Although our search for studies on climate risks, WASH and disability was thorough, limitations may exist due to the strict search strategy. First, climate-related risks are described using varied terminology, and relevant studies may have been missed if key terms were not included in titles or abstracts, or if relevant information appeared only in the full text. Second, qualitative studies, especially those without explicitly mentioning disability in the title or abstract, could have been missed. Third. We excluded papers on water resource management from the search terms, which may have resulted in missing studies on resilient interventions that address competition, conflict, and equity in water access. Forth, the search terms such as ‘menstrual health and hygiene,’ may have introduced a bias toward papers reporting impacts related to cisgender women and girls while overlooking the experiences of other populations that may be vulnerable, including older adults and sexual and gender minorities. Finally, this review was limited to English-language publications and may have overlooked relevant studies and grey literature available in other languages.

## Conclusion

This review underscores the critical intersection between climate change and WASH services, highlighting the disproportionate impact on people with disabilities, women, and girls. Our findings reveal a significant gap in understanding the health consequences of climate-related WASH disruptions, particularly for people with disabilities. The review identifies a pressing need for more comprehensive studies on effective climate-resilient WASH interventions at the household and community levels. The prioritisation of water for agriculture over sanitation and hygiene, coupled with the coping mechanisms employed during extreme weather events, underscores communities’ complex challenges. The review emphasises the need for targeted support mechanisms, sustainable water management practices, and financial and social safety nets to ensure equitable access to WASH services. Substantial efforts are needed to expand the evidence base. This includes rigorous evaluations of climate-resilient WASH technologies and transformative approaches that address underlying social and economic issues. By prioritising disability-inclusive and effective WASH interventions, we can enhance climate resilience and promote health equity for all, particularly the most vulnerable populations.

## Supporting information

S1 Text Working definitions. (DOCX)

S2 Text Water for Women’s Critical Approaches for Climate-Resilient Inclusive WASH Development. (DOCX)

S1 PRISMA Checklist. (DOCX)

S3 Text Search strategy and key terms. (DOCX)

S1 Data Characteristics of included papers. (DOCX)
