# Supplementary material for: Climate change, disability, and water, sanitation and hygiene: A scoping review of evidence and interventions in low and middle-income countries
Source: PLOS Glob Public Health. 2025 Sep 25;5(9):e0003676. doi: 10.1371/journal.pgph.0003676 (PMC12463209; doi:10.1371/journal.pgph.0003676)
Supplement: S1 Text — (DOCX) [file pgph.0003676.s001.docx]

## **Working definitions**

WASH interventions are a set of individual, household or community activities aimed at preventing water-related diseases and enhancing health and wellbeing. They include safely managed water, sanitation and the promotion of effective hygiene behaviours. As set out in the UN Human Rights to Water and Sanitation, interventions should improve the availability of water and sanitation facilities, quality, acceptability, accessibility and affordability [1]. Positive sanitation behaviours include building a latrine and stopping open defecation. Effective hygiene behaviours involve hand hygiene, menstrual health, food hygiene, personal care and safe water collection and storage practices that help maintain health and prevent disease spread.

The Sanitation and Water for All climate task team define climate-resilient WASH services as those that ‘anticipate, respond to, cope with, recover from, adapt to or transform based on climate-related events, trends and disturbances, all while striving to achieve and maintain universal and equitable access to safely managed services, even in the face of an unstable and uncertain climate, where possible and appropriate, minimising emissions, and paying special attention to the most exposed vulnerable groups’ [2].

Achieving climate-resilient WASH necessitates a comprehensive, collaborative approach involving all stakeholders. This includes marginalised groups such as persons with disabilities, women, and girls, as well as households, communities, government entities at all levels, service providers, and regulators. By working together, these stakeholders can effectively identify, assess, and mitigate the risks posed by climate-related events to WASH services and the broader systems that support them. By fostering collaboration, climate-resilient WASH not only safeguards essential services but also plays a pivotal role in strengthening overall community resilience to the impacts of climate change [3].

1. United Nations DoEaSA. Resolution adopted by the General Assembly on 28 July 2010; 64/292. The human right to water and sanitation. Available at <https://digitallibrary.un.org/record/687002?ln=en> (accessed 1 November 2022): 2010.

2. Sanitation and Water for All Climate Task Team. Definition of climate-resilient water sanitation and hygiene services. Available at <https://www.sanitationandwaterforall.org/sites/default/files/2024-11/ClimateResilientWASH_DefinitionPaper_final_0.pdf> (accessed 28 November 2024): 2024.

3. Water for Women. What Does Climate-Resilient Inclusive WASH Look Like? Insights from Water for Women. Available at <https://www.waterforwomenfund.org/en/news/what-does-climate-resilient-inclusive-wash-look-like.aspx> (accessed 17 December 2024): 2024.
