# Supplementary material for: Climate change, disability, and water, sanitation and hygiene: A scoping review of evidence and interventions in low and middle-income countries
Source: PLOS Glob Public Health. 2025 Sep 25;5(9):e0003676. doi: 10.1371/journal.pgph.0003676 (PMC12463209; doi:10.1371/journal.pgph.0003676)
Supplement: S2 Text — (DOCX) [file pgph.0003676.s002.docx]

Water for Women’s Critical Approaches for Climate-Resilient Inclusive WASH Development [1]

| **Three theoretical approaches for addressing climate change effects on WASH services** | **Water for Women’s critical approaches for climate resilient inclusive WASH development** | |
| --- | --- | --- |
| **Theory** | **Critical approach** | **Key insight** |
| *Contextual vulnerability* | Inclusive WASH is essential for climate-resilient WASH development. | Climate-resilient WASH systems are only possible when gender equality, disability and social inclusion principles are applied, ensuring all community members can adapt to and withstand climate events. This requires a transformative approach to gender and social inequalities. |
| *Contextual vulnerability, Resilience* | Adaptable systems and inclusive governance are vital. | Climate risk management must be embedded into existing governance structures, including customary knowledge and climate science. Diverse voices must participate in decision-making to promote sustainable, resilient WASH practices and reduce climate risks for marginalised communities. |
| *Outcome vulnerability, Resilience* | Climate-resilience must be embedded in all phases of the WASH programmes. | WASH programmes should incorporate climate risk and resilience into planning, budgeting, design and monitoring processes from the outset to foster long-term sustainability, adaptability and transformation. |
| *Contextual vulnerability* | Women and marginalised groups offer vital knowledge, capacities and resilience from lived experience. | Women and marginalised groups must be recognised and empowered through transformative approaches that enable greater access and decision-making power. |
| *Outcome vulnerability, Contextual vulnerability, Resilience* | Cross-sector collaboration supports climate-resilient WASH. | Collaboration across sectors on evidence generation and implementation, along with combining public and private financial resources (including climate finance), is essential to meet the needs of vulnerable communities, scale up climate-resilient WASH innovations, and support local adaptive capacities. |

1. Water for Women. What Does Climate-Resilient Inclusive WASH Look Like? Insights from Water for Women. Available at <https://www.waterforwomenfund.org/en/news/what-does-climate-resilient-inclusive-wash-look-like.aspx> (accessed 17 December 2024): 2024.
