# Supplementary material for: Climate change, disability, and water, sanitation and hygiene: A scoping review of evidence and interventions in low and middle-income countries
Source: PLOS Glob Public Health. 2025 Sep 25;5(9):e0003676. doi: 10.1371/journal.pgph.0003676 (PMC12463209; doi:10.1371/journal.pgph.0003676)
Supplement: S1 Data — (DOCX) [file pgph.0003676.s005.docx]

| **Characteristics of included papers** | | | |
| --- | --- | --- | --- |
| **Variable** | **Detail** | **Number** | **%** |
| World Bank Region | East Asia and the Pacific | 1 | 5% |
|  | Latin America and Caribbean | 5 | 23% |
|  | South Asia | 6 | 27% |
|  | Sub-Saharan Africa | 8 | 36% |
|  | Multiple | 2 | 9% |
| Decade of publication | 2015-2019 | 4 | 18% |
|  | 2020-2023 | 18 | 82% |
| Study design type | Qualitative | 8 | 36% |
|  | Quantitative | 4 | 18% |
|  | Mixed-methods | 9 | 41% |
|  | Literature review | 1 | 5% |
| Weather event(s) focused on | Rainfall uncertainty, drought, extreme heat | 9 | 41% |
|  | Increased rainfall and flooding. | 2 | 9% |
|  | Climate change induced sea level rise / groundwater level rise | 1 | 5% |
|  | Rainfall uncertainty and drought; Increased rainfall and flooding. | 7 | 32% |
|  | Rainfall uncertainty and drought; Increased rainfall and flooding; Climate change induced sea level rise / groundwater level rise | 3 | 14% |
| Impact of climate hazards on WASH | Water | 6 | 27% |
|  | Water and Sanitation | 5 | 23% |
|  | Water and hygiene | 5 | 23% |
|  | Sanitation and hygiene | 1 | 5% |
|  | Water, sanitation and hygiene | 5 | 23% |
| Study population focus (including women and girls, and persons with disabilities | General population only | 15 | 39% |
|  | General population, including women and girls | 13 | 59% |
|  | General population, including persons with disabilities | 2 | 9% |
| Theoretical approach(es) used | Outcome vulnerability | 10 | 45% |
|  | Contextual vulnerability | 3 | 14% |
|  | Resilience | 0 | 0% |
|  | Outcome vulnerability; Contextual vulnerability | 6 | 27% |
|  | Outcome vulnerability; Contextual vulnerability; Resilience | 3 | 14% |
